# Analysis of the Region of Interest According to CNN Structure in Hierarchical Pattern Surface Inspection Using CAM

**DOI:** 10.3390/ma14092095

**Published:** 2021-04-21

**Authors:** In Yong Moon, Ho Won Lee, Se-Jong Kim, Young-Seok Oh, Jaimyun Jung, Seong-Hoon Kang

**Affiliations:** Korea Institute of Materials Science, 797, Changwon-daero, Seongsan-gu, Changwon-si, Gyeongsangnam-do 51508, Korea; mooniy085@kims.re.kr (I.Y.M.); h.lee@kims.re.kr (H.W.L.); ksj1009@kims.re.kr (S.-J.K.); oostone@kims.re.kr (Y.-S.O.); jjm0475@kims.re.kr (J.J.)

**Keywords:** class activation map, convolutional neural network, hierarchical pattern, region of interest, surface inspection

## Abstract

A convolutional neural network (CNN), which exhibits excellent performance in solving image-based problem, has been widely applied to various industrial problems. In general, the CNN model was applied to defect inspection on the surface of raw materials or final products, and its accuracy also showed better performance compared to human inspection. However, surfaces with heterogeneous and complex backgrounds have difficulties in separating defects region from the background, which is a typical challenge in this field. In this study, the CNN model was applied to detect surface defects on a hierarchical patterned surface, one of the representative complex background surfaces. In order to optimize the CNN structure, the change in inspection performance was analyzed according to the number of layers and kernel size of the model using evaluation metrics. In addition, the change of the CNN’s decision criteria according to the change of the model structure was analyzed using a class activation map (CAM) technique, which can highlight the most important region recognized by the CNN in performing classification. As a result, we were able to accurately understand the classification manner of the CNN for the hierarchical pattern surface, and an accuracy of 93.7% was achieved using the optimized model.

## 1. Introduction

The surface defect inspection system for initial materials or final products is an important process for quality control and customer satisfaction. Most surface defect inspections are carried out using optical quality control and machine vision [1,2]. Currently, surface defect inspection in many industries is performed by humans using the above technology. However, human inspection at the fast pace of modern industry can cause human errors due to fatigue, which is a financial burden for the company. Automated defect detection (ADD) is applied by categorizing several defects so that the defects can be distinguished from the background of the target surface automatically [3]. Therefore, some major companies are performing defect detection using ADD system. However, this method also has a disadvantage that it is difficult to apply to a surface with a complex background, and a lot of effort by experts is required to select and define the categories. Therefore, the need for a stable and reliable inspection technologies for surfaces with complex backgrounds has emerged.

Recently, with the development of industrial artificial intelligence along with computer technology, many studies have been reported using artificial intelligence to detect surface defects. The convolutional neural network (CNN) is an image-based deep learning algorithm and is a representative model used to surface inspection [4,5,6,7,8,9]. By repetitive training, features that define surface defects are automatically extracted without expert assistance. This method is called an end-to-end method, and if image information is input to the CNN model, it displays good or bad as output. Because the CNN shows outstanding performance in image-based classification problems, it is widely used in various fields such as medical care [10,11], semiconductor [12], manufacturing [13,14], and infrastructure inspection [15]. Representatively, Chen et al. [16] demonstrated that it is possible to detect six kinds of defects on solar cell surface using the CNN. For accurate classification, a multispectral solar cell CNN structure was applied, and it exhibited better performance than the conventional inspection method. In addition, a study reported that combining the Naïve Bayes classifier with the basic CNN structure can effectively perform the surface crack detection [17]. Kim et al. [18] reported that surface defects can be effectively detected by applying weights of the ImageNet model to new problems by transfer learning with fine-tuning.

As described above, the CNN has been accurately applied to surface inspection problems [19,20,21]. However, there is a limitation that the user cannot know what basis or criteria were used for the CNN’s classification due to the nature of the end-to-end model [22]. If we are able to understand the principle of the CNN discrimination, the CNN structure can be elaborately optimized by user. Based on this necessity, an algorithm called the class activation map (CAM) has been recently developed [23,24]. CAM image is a kind of heat-map obtained by adding a global average pooling (GAP) layer at the end of the CNN model. The CAM image can be used to indirectly understand the internal principles of the CNN because it highlights areas that the CNN perceives as the important part of discrimination. Many attempts have been made to understand the classification criteria for the CNN model by using the CAM. Sun et al. [25] developed a model to detect vibrations caused by equipment aging using the CNN combined with the CAM. They showed that artificial intelligence can replace sensor-based fault diagnosis. Additionally, it was revealed that the region where vibration occurred was accurately captured by the CAM. Chen et al. [26] and Li et al. [27] also showed that by applying the CNN together with the CAM for the surface defect inspection, the CNN’s classification criterion can be indirectly understood. CAM images confirmed that various types of defects located locally on the fibers, wood, solar cell and LED chips surfaces are accurately captured and classified. Furthermore, Chen et al. [26] proposed a new spatial attention class activation map (SA-CAM) to improve segmentation adaptability by generating more accurate heat-map. As such, due to the high availability of CAM, the development of surface inspection technology using the CAM is actively progressing.

With increasing interest in multi-functional surfaces, the researches on the fabrication methods for hierarchical patterns have been conducted [28,29]. The representative function of hierarchical patterns is the water-repellent surface, which means that the solid surface repels water. The hierarchical patterns are defined as patterns in which two or more kind of patterns coexist, and generally consist of a macro (or micro) sized primary pattern and a micro (or nano) sized secondary pattern [30]. Recently, we presented that when the hierarchical pattern is formed on the aluminum surface by imprinting using pattern mold produced by electric discharge machining (EDM), the hydrophobicity can be imparted to the aluminum surface [31]. At this time, the hierarchical pattern formed on the aluminum surface consists of a regular primary pattern and an irregular secondary pattern. These complex surface structures are a major obstacle that causes considerable difficulty in discriminating between background and defects.

In this study, a surface with hierarchical patterns was fabricated through the imprinting process. The CNN model was designed to identify surface defects in the fabricated specimens. In order to optimize the CNN architecture, CAM images were used to elaborately analyze the change of the CNN classification manner according to the number of layers and kernel size. Accordingly, the structure could be optimized based on our in-depth understanding of the model. In addition, due to the hierarchical characteristics of the specimen surface, it was possible to analyze the region of interest in the CNN discrimination according to the geometric characteristics of each class and the changes in the model structure. To the best of our knowledge, this is the first research which solves and analyzes the defect inspection for hierarchical pattern surface using the CNN coupled with the CAM approach. As a result, the accuracy of up to 93.7% was achieved using a precisely optimized CNN model.

## 2. Methods

### 2.1. Imprinting Process

**Figure 1:**
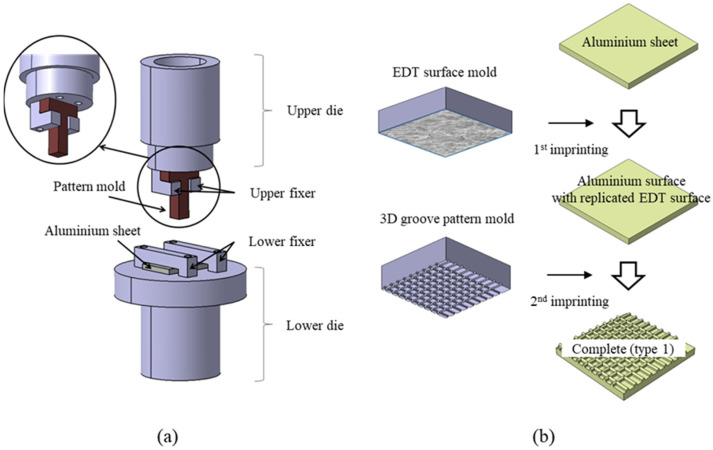
Schematics depicting (**a**) imprinting die-set and (**b**) two-stage imprinting processes.

Figure 1a shows the set of dies used in the imprinting system. The die-set consists of upper and lower dies. A 10 mm × 10 mm pattern mold and a 20 mm × 40 mm aluminum specimen with a thickness 2 mm are fixed to the upper and lower dies, respectively. By jogging the upper die, the pattern mold can press the aluminum specimen. As a result, the structure of the pattern mold is replicated on the aluminum surface.

Figure 1b shows the sequence of the imprinting process. The imprinting is performed in two steps. In the 1st imprinting, the rough electrical discharge texture (EDT) surface is duplicated on the aluminum surface using an EDT surface mold. Thereafter, the groove pattern is replicated using a 3D groove pattern mold in the 2nd imprinting. Through these imprinting processes, the hierarchical pattern is formed on the aluminum surface. Here, the EDT surface mold is the mold in which a rough discharge surface is formed on the mold surface fabricated by EDM, and the 3D groove pattern mold refers to the mold in which the groove pattern is formed on the mold surface fabricated by wire electrical discharge machining (WEDM). This study was carried out by applying a 3D groove pattern mold having a pitch of 600 µm. Detailed information on each mold shape and process can be found in the reference [29].

**Figure 2:**
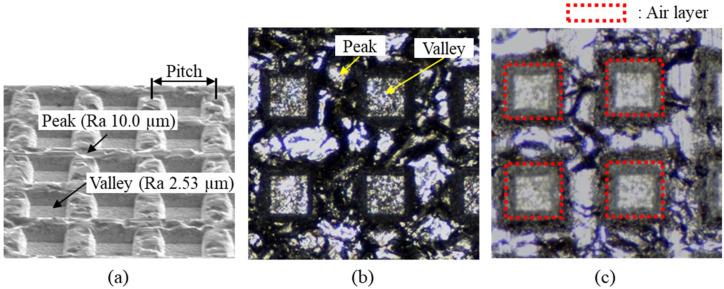
Imprinted aluminum surface: (**a**) measured by SEM, (**b**) measured by optical microscope. (**c**) is a photograph taken when the aluminum specimen is put in water.

Figure 2 shows the surface of the aluminum specimen fabricated through imprinting. As shown in Figure 2a, the pattern surface is divided into peaks and valleys, and each surface roughness was measured as Ra equal to 10.0 µm and 2.5 µm, respectively. The peak regions that the replicated EDT surface formed in the 1st imprinting are maintained even after the 2nd imprinting, and have a rougher morphology than the valley. This is because the rough surface is effective in increasing the water repellency, so that the duplicated EDT surface is deliberately maintained. Therefore, if the replicated EDT surface on the peak region is crushed due to excessive pressing during the 2nd imprinting, the water repellency decreases. Figure 2b,c shows a photograph of the specimen surface taken with an optical microscope, and Figure 2c is taken when the specimen is placed in water. It was found that the air layer is formed in the valley regions. The stable formation of air layer has a great influence on the increase of water repellency, and is a major factor that makes it possible to use the prepared water repellent surfaces for various applications such as anti-icing, anti-fouling, drag-reduction, etc.

**Figure 3:**
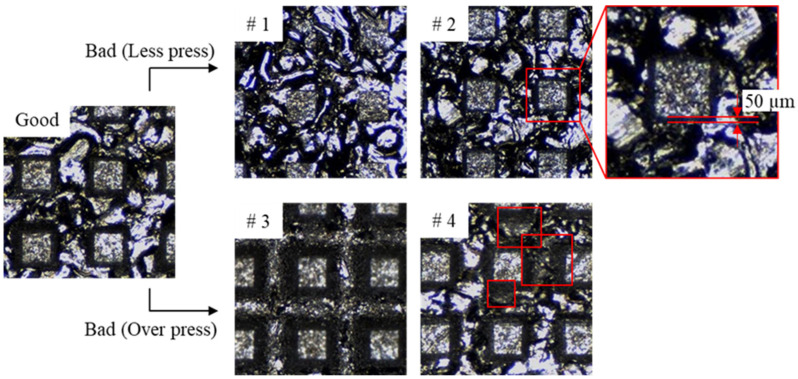
Classification criteria between good and bad specimens during human labeling.

Figure 3 shows three types of the imprinted specimens. If too little or much pressure is applied during the 2nd imprinting, the groove pattern is not sufficiently formed (less press, # 1, 2), or the replicated EDT surface on the peak is crushed (over press, # 3, 4). In addition, when the parallelism between the specimen and the pattern mold does not match during imprinting, or if there is a problem such as foreign matter or damage on the pattern mold surface, the above two defects may occur locally. In general, such defects are inspected by humans using a microscope. However, it is difficult to perform uniform inspection due to the ambiguity of the defect characteristics and human error.

The CNN is a representative data driven model of the artificial intelligence that performs feature extraction by itself. Thus, precisely prepared dataset is crucial to enhance classification ability. Therefore, labeling of the surface images should be performed by human with uniform criteria. From Figure 3, it can be clearly judged that #1 and #3 are less and over press defects. On the other hand, there are ambiguous cases such as #2 and #4 compared to good products. Therefore, the author labeled as less press when the dark area between the peak and valley connection is less than 50 µm. In the case of the over press, if the replicated EDT surface of the peak region is crushed as shown in #4, it was labeled as over press.

One of the most interesting points in this paper is to check whether the CNN’s classification criteria are the same as human labeling, or not the same but use a higher level or simpler manner.

### 2.2. Defect Detection Method Based on CNN with CAM

The CNN is the end-to-end model that performs feature extraction and classification by itself through image-based learning. When a bundle of images is inserted as input data, the kernels with specific size convolute the image horizontally and vertically. Thereafter, they extract and deliver the calculated information to the next layer. This process is repeated for the number of layers to perform classification.

After each convolutional layer, the results are transferred to the next layer in a form of differentiable structure activated by a non-linear activation function. Frequently used activation functions include sigmoid, hyperbolic tangent, and ReLu. In this study, the ReLu function, which is known to be the most effective in image classification, was used [32]. The expression of the related function is as follows.

(1) $$y=\{\begin{array}{c} x,\;x\geq 0\\ 0,\;x<0 \end{array}$$

In the 3rd, 5th, and 6th layers, the maxpooling layers were applied to reduce the data size and improve classification capability in this study. To prevent excessive disappearance of input information, the maxpooling layer was not used in the 1st, 2nd, and 4th layers.

**Figure 4:**
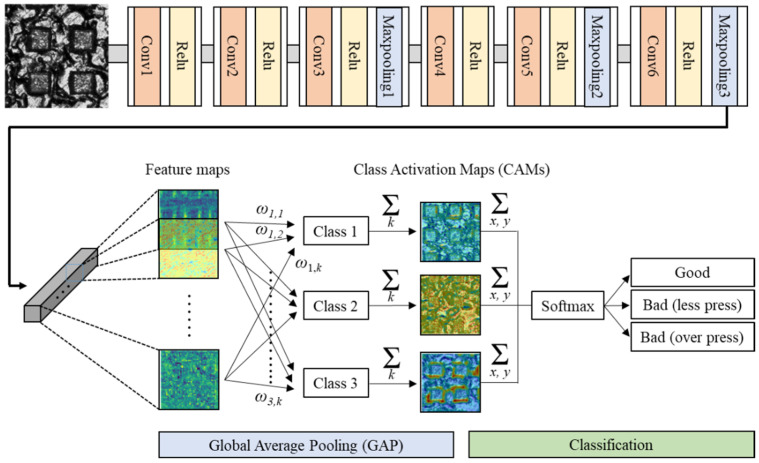
Structure of CNN with CAM.

Since the CNN model is the end-to-end model, only the input information and classification results are known to the user. The internal feature extraction information and classification process are hidden like a black box [24]. Hence, the assessment of CNN’s classification ability can be done simply with some evaluation metrics without information indicating where the CNN recognized as important. In order to improve these shortcomings, the CAM has been recently developed, and many related applications are being studied. The CAM images can be obtained by adding the GAP layer instead of the final fully-connected layer of the general CNN structure. As shown in Figure 4, the information (feature map in the figure) that was generally flattened and transferred to the dense layer is transferred to the GAP layer without being flattened. The transferred information is kept in the form of two-dimensional data and displayed like heat-map after some post-processing. Using the CAM images, we can highlight the region that the CNN recognizes as important in performing classification so that the user can indirectly understand the criteria for the CNN’s determination.

The principle of CAM is as follows. When there are *k* maps from the final convolutional layer, a *ω* value per class is mapped to each feature map. The *ω* also learned through deep learning. When there are *k* feature maps from the final convolutional layer and 3 classes in output, the number of *ω* is *k* × 3. If the result of multiplying values of the feature map and the *ω* are added to all unit *k*, the CAM corresponding to each class is calculated. After that, all values of the CAM are added to make a single number and then classified by putting it in the softmax classifier. Accordingly, the CAM image corresponding to the classification result indirectly reflects the region of interest to identify classes. The related formula is as follows.

(2) $$S_{c}=\underset{k}{\sum }\omega_{k}^{c}\underset{x,y}{\sum }f_{k}\left(x,y\right)=\underset{x,y}{\sum }\underset{k}{\sum }\omega_{k}^{c}f_{k}\left(x,y\right)$$

(3) $$M_{c}\left(x,y\right)=\underset{k}{\sum }\omega_{k}^{c}f_{k}\left(x,y\right)$$

where *S_c_*, $\omega_{k}^{c}$, *f_k_*(*x*,*y*)*, M_c_*(*x*,*y*) represent the class score for given class *c*, the weight corresponding to class *c* for unit *k*, the feature map of unit k in the last convolution layer at a spatial location (*x*, *y*), and the class activation map for class *c*. The *S_c_* value obtained by Equation (2) is applied to the final softmax classifier, and the equation is defined as follows.

(4) $$P_{c}=\frac{exp\left(S_{c}\right)}{\sum_{c}exp\left(S_{c}\right)}$$

where *P_c_* represents the output probability.

The overall structure of the designed deep learning model was summarized in Table 1. It consists of up to 6 convolutional layers. The kernel size of all convolutional layers is basically designed as 10 by 10. It is noted that the structure will be changed in order to analyze the effect of the number of convolutional layers and kernel size. The input data of 3rd, 5th, and 6th layers was reduced by half by designing the kernel size of the maxpooling layer to be 2 by 2. The *Nx* and *Ny* dimension values of the output shape in the final GAP layer are the same as the *x* and *y* dimensions of the output shape in the previous layer. The *k* dimension of output shape in the GAP layer is 3 because there are three classes (good, less press, over press).

**Figure 5:**
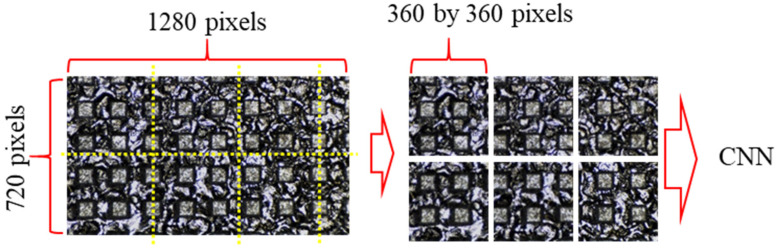
Preparation of input images by cropping an original image.

Figure 5 shows how to prepare the raw image as input images to be applied to the model. The initial image at 1280 by 720 pixels was cropped to 360 by 360 pixels. The cropped images were used as input images to reduce computation time and memory consumption. This pre-processing is considered to have no effect on model training because the surface has a regularly repeated pattern. In addition, there is the advantage in reducing over-fitting problem by increasing the amount of dataset to use for model training. The number of prepared input images is shown in Table 2. The ratio of the training dataset to the test dataset was set to 8:2. The amount of good, less press, and over press was prepared at the same ratio so that the amount of dataset did not affect each classification ability.

### 2.3. Evaluation Metrics

In order to evaluate the classification ability in detail, accuracy, precision, and recall values were used in this study. To calculate each value, the factors of true positive (TP), fault positive (FP), fault negative (FN), and true negative (TN) were used. The meaning of each is shown in Table 3. For example, TP refers to the number of good surfaces that are accurately judged to be good. Using the obtained TP, FP, FN, and TN values, the accuracy, precision, and recall are calculated through Equations (5)–(7) [33,34]. The accuracy means ratio of correct predictions over the total number of instances evaluated. On the other hand, the precision and recall indirectly represent the ability to accurately detect defects and the ability to accurately detect good surfaces, respectively.

(5) $$Accuracy=\frac{TP+TN}{TP+FP+TN+FN}$$

(6) $$Precision=\frac{TP}{TP+FP}$$

(7) $$Recall=\frac{TP}{TP+FN}$$

In addition to the commonly used evaluation metrics above, true less press_rate (TL_rate) and true over press_rate (TO_rate) were used to individually analyze the detectability of the less press and the over press. TL_rate means the ratio accurately predicted as less press among the images labeled as less press. TO_rate refers to the ratio accurately predicted as over press among the images labeled as over press. By adding these values, it was possible to analyze whether there is a difference in the detectability of the two kinds of defects when the structure of the model is changed.

## 3. Results and Discussion

### 3.1. Effect of the Number of Convolutional Layers

Five kinds of the CNN models composed of 2 to 6 layers were trained to evaluate the classification ability according to the number of layers. The kernel size of all convolutional layers is fixed at 10 by 10. The training was performed using the deep learning model package embedded in TensorFlow. The optimization was performed using AdamOptimizer with a learning rate of 0.0001. The model was trained for 5000 epochs and 10 mini-batches.

**Figure 6:**
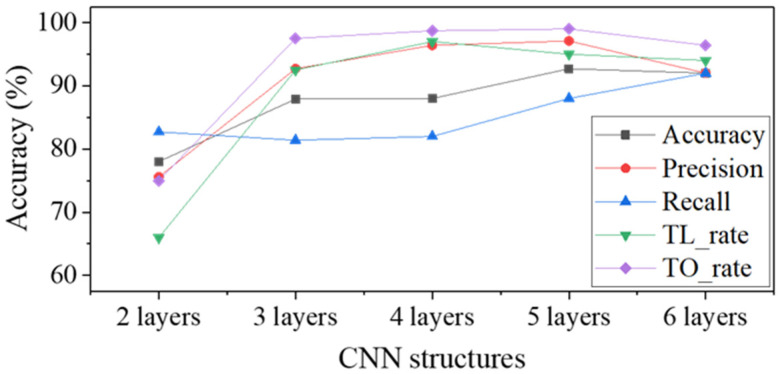
Graph depicting the defect inspection results according to the number of layers.

Table 4 and Figure 6 show the results of inspection. By analyzing the evaluation metrics, three main trends can be identified. First, the TO_rate is higher than the TL_rate in all the number of layers. In other words, over press detection is better than less press detection. Second, based on the accuracy, precision and recall metrics, these values increase as increasing the depth of the CNN, so that they reach maximum values at 5 layers, and it tends to decrease when it reaches 6 layers (The recall value is highest at 6 layers). Third, the precision values tend to be higher or equal to the recall values except for the 2 layers. This means that the ability to judge a defective surface as a defect is better than the ability to judge a good surface as good. The reason for the difference in classification ability according to the type of classes can be interpreted through CAM images.

**Figure 7:**
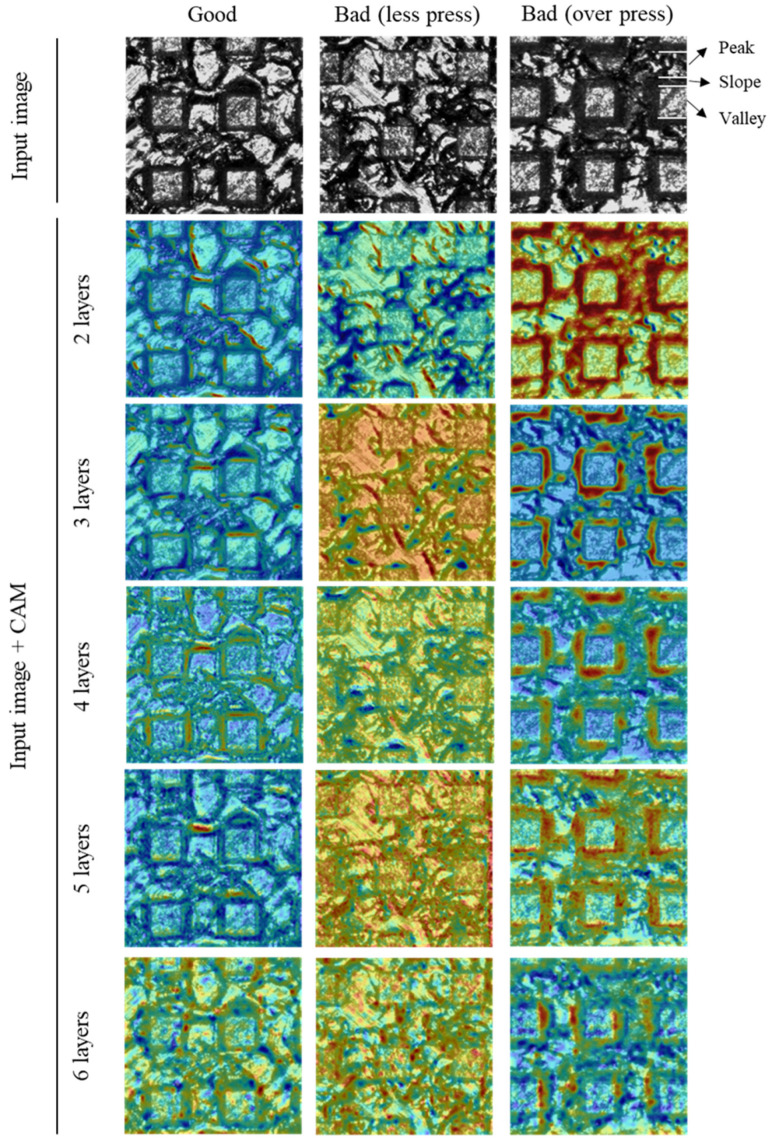
Changes in regions considered to be important in determining the CNN according to the number of layers.

Figure 7 shows the CAM images according to the depth of the CNN model. For accurate comparative analysis, CAM images for the same input image are presented. In the figures, the region highlighted in red represents the important region for the CNN classification. The interpretation using CAM for the three trends mentioned above are listed below.

#### 3.1.1. The Reason Why TO_Rate Is Higher Than TL_Rate

The result in which TO_rate is higher than TL_rate can be explained by checking the CAM image of 5 layers. In the case of the over press class, the CNN model mainly paid attention to the dark region of the slope where the peak and valley are connected, and relatively less attention was paid to the peak region (The dark area of the slope is a feature that is uniformly observed in over press labeled images, and was not used as a criterion for labeling by human. Therefore, it is meaningful criterion that learned by itself through feature learning). Therefore, it can be interpreted that the over press defect is determined by identifying the features of the local region. On the other hand, less press class are paid attention to the entire surface area. This means that less press is identified based on the information from global regions. The difference in accuracy is judged to occur depending on whether the criteria for determining the two classes are local or global. Therefore, the reason why TO_rate is higher than TL_rate is thought to be because the over press has a locally clear and noticeable feature.

Additionally, for the less press class, the CNN model did not consider the dark and narrow slope region, which was the criterion for labeling by human (50 µm or less in width). Nevertheless, TL_rate shows a high value of about 95%. This means that the CNN model has learned that the entire information including the peak and valley is a better criterion for identifying the defects by less press.

#### 3.1.2. The Effect of CNN Model Depth

In the case of the two layers, there is a slight difference between good and less press CAM images. The low accuracy (78%) and TL_rate (66%) values were measured. In the case of the over press, the region where the crushed EDT surface of the peak and the slope area are accurately paid attention. However, since the other regions are also slightly highlighted, the classification cannot be accurately performed and TO_rate was measured at 75%. When the depth of CNN increased to 3 layers, a noticeable difference occurs between the CAM images of good and less press. For the good class, a dark area in a thinly formed slope region is used as a criterion for judgment without paying attention to the entire surface. On the contrary, it can be found that less press was classified by paying attention to the entire surface area. This can be interpreted as the CNN model with 3 layers are starting to detect less press defect by paying attention to the entire surface, including the peak and valley. In the case of the over press, the dark areas of slope are clearly paid attention but peak regions are not. This means that from the CNN model with 3 layers, the model noticed that the peak regions do not need to be considered for over press determination. As a result, the TO_rate has increased significantly to 97.5%.

After that, as the depth of CNN increased, the accuracy also increased and reached a maximum 92.7% in 5 layers. The CAM images in 5 layers have no distinct difference from 3 layers. It seems that the weight values were elaborately improved without big changes in the classification criterion between 3 layers and 5 layers. When the depth of CNN reaches to 6 layers, the precision decreased from 97.1% to 92%, and recall increased from 88% to 92%. This means that the ability to accurately classify the defective surfaces has decreased, but the ability to accurately classify the good surfaces has increased. This result can be explained through the CAM image of over press. Unlike the CAM images in the previous number of layers, the highlighted range near the slope is significantly reduced. This is considered to be because the local information around the slope was vanished as the depth of the layers increased. For this reason, TO_rate was reduced from 99% to 96.4% and also the precision decreased.

#### 3.1.3. The Reason Why Precision Is Higher Than Recall

The high precision and low recall reflect that the model is more effective in detecting defects than detecting good surfaces. This trend can be explained through CAM images. As shown in 5 layers CAM images, the range and intensity of the highlighted region on the good surface are smaller and weaker than the other two defective surfaces. This is believed to be due to the fact that less press and over press defects have clear characteristics such as entire surface information for less press and slope range for over press, while good surface has small and faint features that are difficult to distinguish. Moreover, complex surface morphology by hierarchical pattern seems to make the detection of good surface more difficult. As will be described in Section 3.2, this problem can be improved by reducing the kernel size. Through the analysis on depth of model, it was found that the CNN model with 5 layers shows the best classification ability.

### 3.2. Effect of the Kernel Size

**Figure 8:**
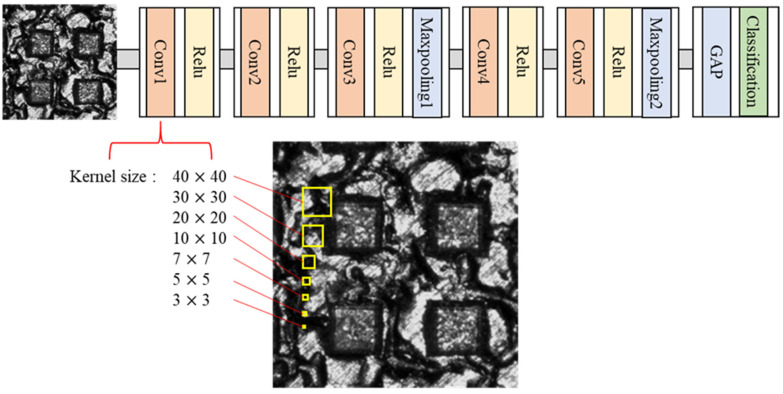
Modification of kernel size at the 1st convolutional layer.

In general, when the kernel size increases, more surrounding information of input data are extracted [16]. Accordingly, the kernel size in the first convolutional layer has a great influence on the characteristics of extracting information of the initial input image. Therefore, the kernel size of the 1st convolutional layer was designed in various sizes as shown in Figure 8. It can be seen that the smallest 3 by 3 kernel is smaller than the dark region between peak and valley. The largest 40 by 40 kernel is large enough to occupy half of the peak. Therefore, it makes possible to know how these initial kernel characteristics affect inspection results. After training the modified CNN model with 5 layers, the classification was performed.

**Figure 9:**
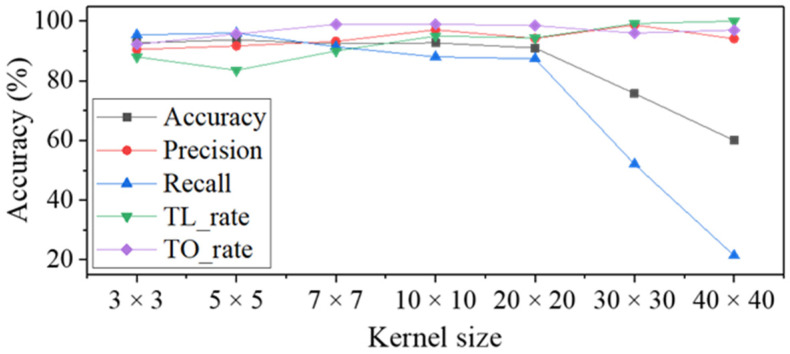
Graph depicting the defect inspection results according to the kernel size of 1st layer.

**Figure 10:**
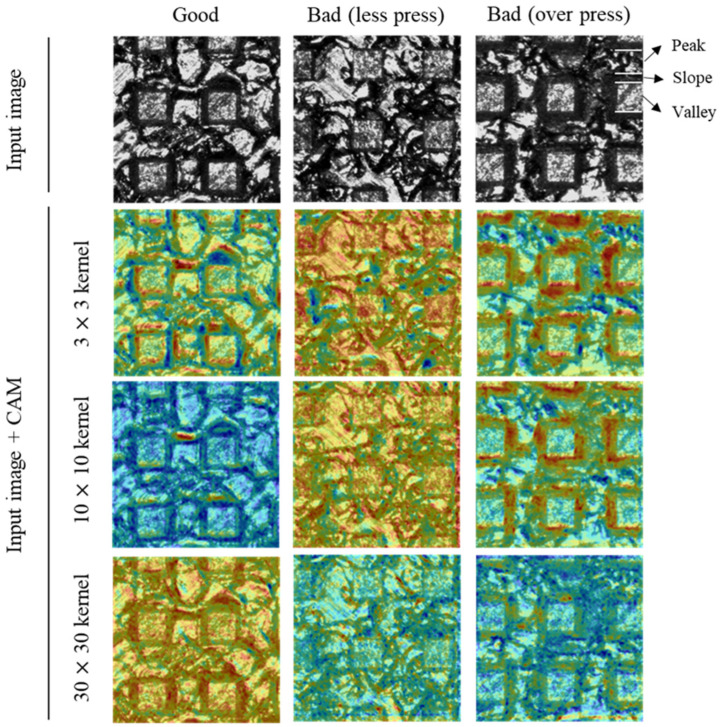
Changes in regions considered important in determining CNN according to the kernel size at 1st layer.

Table 5 and Figure 9 show the inspection result. The most noticeable result from the analysis is that when the kernel size increases to 30 by 30 or more, accuracy and recall dramatically decrease but the precision increases. This trend means that increasing the kernel size is helpful in detecting defective surfaces. However, it has a negative effect on accurately determining good surfaces. The cause of these results can be explained through the CAM image in Figure 10. As can be seen in good class of the 30 by 30 kernel, although there is a difference in intensity, both of the slope and the entire surface are highlighted in red at the same time. This is equivalent to the region of interest for over press and less press of 10 by 10 kernel, respectively. In other words, good surfaces could not be clearly distinguished from the two bad classes. It is considered that this result occurred because the kernel size was so large that it was impossible to extract important local information from the 1st convolutional layer. In addition, the fact that recall shows the highest value at the kernel size of 5 by 5 supports that a small kernel size is advantageous for detecting good surfaces. This is because the dark region of the slope, which the CNN model uses as the criteria for detecting good surfaces, is formed narrowly. It seems that a small size of kernel is better to accurately extract the information of the narrow region of the initial input image. Therefore, the kernel size should not exceed 20 by 20, and 5 by 5 is the most effective kernel size based on accuracy.

### 3.3. Compatibility

**Figure 11:**
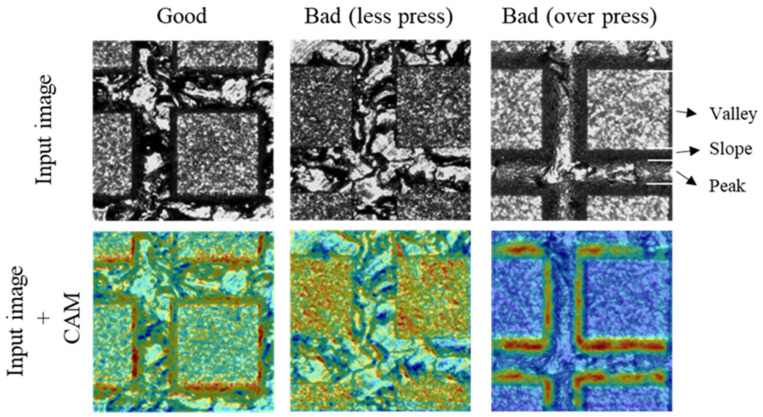
Results of CNN classification using the groove pattern with 1000 µm pitch.

To check whether the trained CNN model identifies the same region of interest for a pattern with different pitch size, the image of groove pattern with 1000 µm in pitch was applied to the model. The results are shown in Figure 11. The CNN model with 5 layers and 5 by 5 kernel for 1st convolutional layer was applied. As shown in CAM images, each class was judged in the same manner described in Section 3.1 and Section 3.2. The good surface and over press defect were detected by paying attention to narrow or wide slope regions, respectively. The less press defect was distinguished based on the entire information including peak and valley by paying attention to the entire surface. It is concluded that the CNN model for inspection of hierarchical patterns with complex backgrounds can be applied to patterns with different pitches.

### 3.4. Comparison with Other CNN Structures

In order to confirm that our CNN with CAM model (5 layers and 5 by 5 kernel for 1st convolutional layer) was optimized completely, a further experiment was conducted using the solar cell CNN structure [16], which was recently introduced in the field of surface inspection. The solar cell CNN, announced in 2020, was designed to apply surface inspection of the solar cells, and it has been demonstrated that it can be effectively applied for surface inspection of wood and metal [26].

The solar cell CNN was designed identically based on the paper [16], and the structure is shown in Table 6. Briefly, it consists of 5 convolutional layers. Two fully-connected layers are connected after 5th convolutional layer. A dropout was performed between the 2nd fully-connected layers and the output layer. The dropout ratio during training was set to 0.5. The optimization was performed using AdamOptimizer with a learning rate of 0.0001. The model was trained for 5000 epochs and 10 mini-batches.

Two additional CNN structures were added for precise comparative analysis. The first is a CNN without CAM model in which the GAP layer is removed from the CNN with CAM model and two fully-connected layers are added in the same way as the solar cell CNN. The second is the solar cell CNN + CAM in which a GAP layer is added instead of two fully-connected layers in the solar cell CNN. By adding those CNN models for our comparison study, we tried to analyze not only the effect of the convolutional parameters (kernel size), but also the effect of the fully-connected layers.

Table 7 shows the inspection results of the four kinds of CNN models. As can be seen, both the solar cell CNN and the CNN without CAM, which has fully-connected layers, showed remarkably low accuracy. It is believed that the inspection ability was degraded because the location information of the defect region disappeared due to the fully-connected layer. For example, when detecting a simple scratch (or defect) on a wood surface, it can be detected by checking the scratch on the entire surface. However, the classification in the hierarchical pattern requires not only presence or absence of defect region, but also the location information indicating the location of the defect. For this reason, the use of the fully-connected layer should be avoided in our surface inspection. When the GAP layer was added to solar cell CNN instead of the fully-connected layers, the accuracy increased from 46.4 to 76.4% compared to the basic solar cell CNN. However, this is a significantly lower value compared to the result of the CNN with CAM. These low accuracy of the solar cell CNN, which showed high accuracy for wood, metal, and solar cell surfaces, is thought to be due to the lack of optimization of the CNN structure such as kernel size and the number of layers. In other words, even if there is an existing model with good performance, the hyperparameters of the model should be carefully optimized to inspect the pattern surface with hierarchical structure introduced in this study. Therefore, it can be concluded that the parameter optimization of our CNN with CAM model was performed well.

## 4. Conclusions

In this paper, we proposed the CNN with CAM model for surface inspection of the hierarchical patterned surface with complex background. The optimization of the CNN architecture was analyzed in detail using the CAM images along with several evaluation metrics. Through this, it was possible to strictly analyze how the structure of the model represented by the number of layers and kernel size affects the feature extraction performance. In addition, it was demonstrated that an in-depth understanding of CNN’s criterion can suggest the direction of CNN structure optimization. In summary, it was confirmed that the CNN with CAM model paid attention to the unique characteristics representing each class. When the characteristics of small region play an important role in determining the classes, a small kernel size is effective. In the opposite case, it is effective to extract the surrounding information by increasing the kernel size. In addition, by changing the number of layers of the CNN model, it was confirmed how the criteria for discriminating each class changed. Furthermore, based on CAM images, it was found that the classification was performed by more effective criteria than the labeling criteria by humans. These results show the possibility that the CNN with CAM can be used to extract features of defective products. With respect to the future research, the target surface applied in this study has not many kinds of classes, so there was no great difficulty in classification. Therefore, it is thought that the classification problem for hierarchical patterns with many types of classes will be a good research direction.

## Figures and Tables

**Table 1:** Structure and parameters of CNN with CAM.

Name	Structures	Kernel Size	Channel	Output Shape (*x* × *y* × *k*)
Input image	-	-	1 (gray)	360 × 360 × 1
1st layer	Conv 1	10 × 10	64	360 × 360 × 64
2nd layer	Conv 2	10 × 10	64	360 × 360 × 64
3rd layer	Conv 3	10 × 10	64	360 x 360 x 64
	Max pooling 1	2 × 2	-	180 × 180 × 64
4th layer	Conv 4	10 × 10	64	180 × 180 × 64
5th layer	Conv 5	10 × 10	32	180 × 180 × 64
	Max pooling 2	2 × 2	-	90 × 90 × 32
6th layer	Conv 6	10 × 10	32	90 × 90 × 32
	Max pooling 3	2 × 2	-	45 × 45 × 32
GAP	-	-	-	N*_x_* × N*_y_* × 3

**Table 2:** The type and number of dataset.

Type	Training Set	Test Set	Sum
Good	1118	271	1389
Bad (less press)	1133	276	1409
Bad (over press)	1109	293	1402
Total	3360	840	4200

**Table 3:** Parameters to evaluate inspection results.

-		Actual Condition	
		Normal	Fault
Predicted condition	Normal	TP	FP
	Fault	FN	TN

**Table 4:** Defect inspection results according to the number of layers.

No. of Layers	Accuracy (%)	Precision (%)	Recall (%)	TL_Rate (%)	TO_Rate (%)
2 layers	78	75.6	82.7	66	75
3 layers	87.9	92.7	81.4	92.5	97.5
4 layers	88	96.4	82	97	98.7
5 layers	92.7	97.1	88	95	99
6 layers	92	92	92	94	96.4

**Table 5:** Defect inspection results according to kernel size of 1st layer.

Kernel Size	Accuracy (%)	Precision (%)	Recall (%)	TL_Rate (%)	TO_Rate (%)
3 × 3	92.7	90.5	95.4	88	92.2
5 × 5	93.7	91.7	96	83.5	95.7
7 ×7	92.4	93.2	91.4	90	98.9
10 × 10	92.7	97.1	88	95	99
20 × 20	91	94.2	87.4	94.4	98.5
30 × 30	75.7	98.7	52	99.2	96
40 × 40	60	94.1	21.4	100	97

**Table 6:** Architecture of the solar cell CNN. Adapted with permission from ref. [16].

Name	Structures	Kernel Size	Channel	Output Shape (*x* × *y* × *k*)
Input image	-	-	1 (gray)	360 × 360 × 1
1st layer	Conv 1	7 × 7	16	360 × 360 × 16
	Max pooling 1	2 × 2	-	180 × 180 × 16
2nd layer	Conv 2	5 × 5	32	180 × 180 × 32
3rd layer	Conv 3	5 × 5	32	180 × 180 × 32
	Max pooling 2	2 × 2	-	90 × 90 × 32
4th layer	Conv 4	3 × 3	64	90 × 90 × 64
5th layer	Conv 5	3 × 3	64	90 × 90 × 64
	Max pooling 3	2 × 2	-	45 × 45 × 64
6th layer	Fully-connected 1	-	-	512
	Fully-connected 2	-	-	512
Output	-	-	-	3

**Table 7:** Different results according to CNN architectures.

Training Models	Accuracy (%)	Precision (%)	Recall (%)
CNN with GAP	93.7	91.7	96.0
CNN without GAP	24.7	29.3	36.0
Solar cell CNN [16]	46.4	48.1	90.7
Solar cell CNN [16] + GAP	76.4	94.4	56.0

## Data Availability

The data presented in this study are available on request from the corresponding author. The data are not publicly available due to the Korea Institute of Materials Science (KIMS) software management regulations.

## References

[1] Dhillon A., Verma G.K. (2020). Convolutional neural network: A review of models, methodologies and applications to object detection. Prog. Artif. Intell..

[2] Wang T., Chen Y., Qiao M., Snoussi H. (2018). A fast and robust convolutional neural network-based defect detection model in product quality control. Int. J. Adv. Manuf. Technol..

[3] Weimer D., Scholz-Reiter B., Shpitalni M. (2016). Design of deep convolutional neural network architectures for automated feature extraction in industrial inspection. CIRP Ann..

[4] Ji Y., Zhang H., Zhang Z., Liu M. (2021). CNN-based encoder-decoder networks for salient object detection: A comprehensive review and recent advances. Inf. Sci..

[5] Jiao J., Zhao M., Lin J., Liang K. (2020). A comprehensive review on convolutional neural network in machine fault diagnosis. Neurocomputing.

[6] Słoński M., Schabowicz K., Krawczyk E. (2020). Detection of flaws in concrete using ultrasonic tomography and convolutional neural networks. Materials.

[7] Wang W., Shi P., Deng L., Chu H., Kong X. (2020). Residual Strength Evaluation of Corroded Textile-Reinforced Concrete by the Deep Learning-Based Method. Materials.

[8] Słoński M., Tekieli M. (2020). 2D digital image correlation and region-based convolutional neural network in monitoring and evaluation of surface cracks in concrete structural elements. Materials.

[9] Liu Y., Yuan Y., Balta C., Liu J. (2020). A Light-Weight Deep-Learning Model with Multi-Scale Features for Steel Surface Defect Classification. Materials.

[10] Anthimopoulos M., Christodoulidis S., Ebner L., Christe A., Mougiakakou S. (2016). Lung pattern classification for interstitial lung diseases using a deep convolutional neural network. IEEE Trans. Med. Imaging..

[11] Yamashita R., Nishio M., Do R.K.G., Togashi K. (2018). Convolutional neural networks: An overview and application in radiology. Insights Imaging.

[12] Nakazawa T., Kulkarni D.V. (2018). Wafer map defect pattern classification and image retrieval using convolutional neural network. IEEE Trans. Semicon. Manuf..

[13] Wang Y., Liu M., Zheng P., Yang H., Zou J. (2020). A smart surface inspection system using faster R-CNN in cloud-edge computing environment. Adv. Eng. Inform..

[14] Wang J., Ma Y., Zhang L., Gao R.X., Wu D. (2018). Deep learning for smart manufacturing: Methods and applications. J. Manuf. Syst..

[15] Kumar S.S., Abraham D.M., Jahanshahi M.R., Iseley T., Starr J. (2018). Automated defect classification in sewer closed circuit television inspections using deep convolutional neural networks. Autom. Constr..

[16] Chen H., Pang Y., Hu Q., Liu K. (2020). Solar cell surface defect inspection based on multispectral convolutional neural network. J. Intell. Manuf..

[17] Chen F.C., Jahanshahi M.R. (2017). NB-CNN: Deep learning-based crack detection using convolutional neural network and Naïve Bayes data fusion. IEEE Trans. Ind..

[18] Kim S., Kim W., Noh Y.K., Park F.C. Transfer learning for automated optical inspection. Proceedings of the 2017 International Joint Conference on Neural Networks (IJCNN).

[19] Zhou Z., Lu Q., Wang Z., Huang H. (2019). Detection of micro-defects on irregular reflective surfaces based on improved faster R-CNN. Sensors.

[20] Sun X., Gu J., Huang R., Zou R., Giron Palomares B. (2019). Surface defects recognition of wheel hub based on improved faster R-CNN. Electronics.

[21] Nguyen T.P., Choi S., Park S.J., Park S.H., Yoon J. (2021). Inspecting method for defective casting products with convolutional neural network (CNN). Int. J. Precis. Eng. Manuf. Green Tech..

[22] Park J.K., Kwon B.K., Park J.H., Kang D.J. (2016). Machine learning-based imaging system for surface defect inspection. Int. J. Precis. Eng. Manuf. Green Tech..

[23] Zhou B., Khosla A., Lapedriza A., Oliva A., Torralba A. Learning deep features for discriminative localization. Proceedings of the IEEE Conference on Computer Vision and Pattern Recognition.

[24] Selvaraju R.R., Cogswell M., Das A., Vedantam R., Parikh D., Batra D. Grad-cam: Visual explanations from deep networks via gradient-based localization. Proceedings of the IEEE International Conference on Computer Vision (ICCV).

[25] Sun K.H., Huh H., Tama B.A., Lee S.Y., Jung J.H., Lee S. (2020). Vision-based fault diagnostics using explainable deep learning with class activation maps. IEEE Access.

[26] Chen H., Hu Q., Zhai B., Chen H., Liu K. (2020). A robust weakly supervised learning of deep Conv-Nets for surface defect inspection. Neural. Comput. Appl..

[27] Lin H., Li B., Wang X., Shu Y., Niu S. (2019). Automated defect inspection of LED chip using deep convolutional neural network. J. Intell. Manuf..

[28] Simpson J.T., Hunter S.R., Aytug T. (2015). Superhydrophobic materials and coatings: A review. Rep. Prog. Phys..

[29] Zhang X., Shi F., Niu J., Jiang Y., Wang Z. (2008). Superhydrophobic surfaces: From structural control to functional application. J. Mater. Chem..

[30] Moon I.Y., Kim B.H., Lee H.W., Oh Y.S., Kim J.H., Kang S.H. (2020). Superhydrophobic polymer surface with hierarchical patterns fabricated in hot imprinting process. Int. J. Precis. Eng. Manuf. Green Technol..

[31] Moon I.Y., Kang S.H., Yoon J. (2021). Hydrophobic aluminum alloy surfaces fabricated by imprinting process and their wetting state evaluation using air layer images. Int. J. Precis. Eng. Manuf..

[32] Glorot X., Bordes A., Bengio Y. Deep sparse rectifier neural networks. Proceedings of the Fourteenth International Conference on Artificial Intelligence and Statistics.

[33] Powers D.M. (2020). Evaluation: From precision, recall and F-measure to ROC, informedness, markedness and correlation. arXiv.

[34] Hossin M., Sulaiman M.N. (2015). A review on evaluation metrics for data classification evaluations. Int. J. Data Min. Knowl. Manag. Process.

